# Radial endobronchial ultrasound with a guide sheath for diagnosis of peripheral cavitary lung lesions: a retrospective study

**DOI:** 10.1186/s12890-016-0244-y

**Published:** 2016-05-11

**Authors:** Manabu Hayama, Norio Okamoto, Hidekazu Suzuki, Motohiro Tamiya, Takayuki Shiroyama, Ayako Tanaka, Takuji Nishida, Takashi Nishihara, Nobuko Uehara, Naoko Morishita, Kunimitsu Kawahara, Tomonori Hirashima

**Affiliations:** Department of Thoracic Malignancy, Osaka Prefectural Medical Center for Respiratory and Allergic Diseases, 3-7-1, Habikino, Habikino City, Osaka 583-8588 Japan; Department of Pathology, Osaka Prefectural Medical Center for Respiratory and Allergic Diseases, Osaka, Japan

**Keywords:** Radial endobronchial ultrasound, Endobronchial ultrasound with a guide sheath, Lung cancer, Cavity, peripheral lung lesion, Transbronchial biopsy, Bronchoscopy

## Abstract

**Background:**

Radial endobronchial ultrasound with a guide sheath (EBUS-GS) has improved the diagnostic outcomes of peripheral lung lesions. However, to our knowledge, reports on the use of EBUS-GS for diagnosis of cavitary lesions are unavailable. Therefore, this study aimed to assess the effectiveness and safety of EBUS-GS for diagnosis of peripheral cavitary lung lesions (PCLLs).

**Methods:**

This study was a single-institution retrospective review of PCLLs examined by using EBUS-GS between July 2013 and October 2015. The diagnostic results of different EBUS-GS samples, including cytologic, histopathologic, and microbiologic samples, were analysed separately.

**Results:**

Of 696 radial EBUS procedures performed during the study period, 50 were performed for examination of PCLLs. The overall diagnostic yield for EBUS-GS was 80 % (40/50). Regarding 27 malignant lesions, the diagnostic yields for cytologic and histopathologic samples were 63.0 % (17/27) and 74.1 % (20/27), respectively. Regarding 23 benign lesions, the diagnostic yields for histopathologic and microbiologic samples were 69.6 % (16/23) and 47.8 % (11/23), respectively. Uni- and multivariate analyses indicated that the EBUS probe being within the lesion was the only factor significantly associated with increased diagnostic yield (odds ratio, 7.04; *P* = 0.03). Although pulmonary infection occurred after the procedure in 1 patient (2.0 %), no other complications, including pneumothorax or significant haemorrhage, were reported.

**Conclusion:**

EBUS-GS was found to be an effective and safe procedure for diagnosis of PCLLs.

## Background

A pulmonary cavity is defined as a gas-filled area within a pulmonary consolidation, mass, or nodule that may or may not contain a fluid level and is surrounded by a wall, usually of varied thickness [1]. Various malignant and benign diseases can present as cavitary lung lesions. Because findings on chest radiography and computed tomography (CT) often overlap between malignant and benign cavitary lesions, minimally invasive specimen collection is often necessary for accurate diagnosis [2]. However, sufficient diagnostic biopsy samples may not be obtained due to the limited target area of the cavity wall and the surrounding reactive normal tissue [3].

According to the guidelines of the American College of Chest Physicians, the sensitivity of transbronchial biopsy (TBB) for diagnosis of peripheral lung lesions is 63 % for lesions >20 mm, and only 34 % for lesions <20 mm [4]. On the other hand, the pooled sensitivity of transthoracic needle aspiration (TTNA) for diagnosis of peripheral lung cancer is 90 %, making it the preferred method for preoperative diagnosis [4]. However, TTNA carries a high risk of complications, such as pneumothorax, air embolism, and tumour seeding [5, 6]. With regard to bronchoscopy for diagnosis of peripheral lung lesions, a recent meta-analysis reported a diagnostic yield of 70 % with use of electromagnetic navigation bronchoscopy, radial endobronchial ultrasound (R-EBUS) with or without a guide sheath, virtual bronchoscopic navigation (VBN), and ultrathin bronchoscopes; among these, the pooled diagnostic yield was highest with use of a guide sheath [7]. Therefore, because of its safety profile and sufficient diagnostic yield, R-EBUS with a guide sheath (EBUS-GS) is now becoming a common procedure for examination of peripheral lung lesions [8].

However, reports on the use of R-EBUS for diagnosis of cavitary lung lesions with a relatively high probability of infectious disease are limited [9]. Because it is unknown whether a lung lesion is malignant or benign before diagnostic bronchoscopy, malignant and benign lesions usually are examined by using the same procedure [10]. The aim of this study was to assess the effectiveness and safety of EBUS-GS for diagnosis of peripheral cavitary lung lesions (PCLLs).

## Methods

### Study approval

This study was a retrospective review of a prospectively maintained database and medical records at our institution. The Institutional Review Board of Osaka Prefectural Medical Center for Respiratory and Allergic Diseases approved this study without the need to obtain informed consent. Written informed consent for bronchoscopy was obtained from all eligible patients.

### Patient selection

Patient information (age, sex, type of procedure) and lesion characteristics were collected prospectively in the database before diagnostic bronchoscopy. Lesion characteristics, including type (solid, part-solid, pure ground-glass opacity [GGO], cavitary, or other [i.e., atelectasis, diffuse or lobar consolidation]), maximum diameter, target bronchus, and presence of bronchus sign, were evaluated on thin-section chest CT images during regular team discussions that included board-certified bronchoscopists, pulmonologists, and medical oncologists. VBN (LungPoint, Bronchus Ltd.) was used when the target bronchus was small and difficult to trace [11].

PCLLs examined by using EBUS-GS at our institution between July 2013 and October 2015 were included in this study. Cases in which ultrathin bronchoscopes (BF-XP260, Olympus Ltd.) were additionally used were excluded. A PCLL was defined as a bronchoscopically invisible lung lesion that had an internal gas-filled area and was surrounded by pulmonary parenchyma [1, 8]. Maximum cavity wall thickness and minimum distance from the costal pleura were additionally assessed on chest CT transverse images.

### EBUS-GS procedure

All EBUS-GS procedures were performed by expert bronchoscopists or trainees with sufficient experience with conventional bronchoscopy and EBUS-GS assistance. During all the procedures, the trainees were supervised by the experts.

EBUS-GS procedures were carried out by using one of the following bronchoscopes (Olympus Ltd.): BF-1T260, BF-P260, BF-F260, or BF-Y0053, a new middle-range bronchoscope with a 5.1-mm outer diameter and a 2.6-mm working channel [12]. A guide sheath kit (K-202 or K-204, Olympus Ltd.) was used in combination with an R-EBUS probe (UM-S20-17S, Olympus Ltd.). All bronchoscopies were performed via an oral route under local anaesthesia with intravenous midazolam for mild sedation.

Upon insertion of the scope to reach the target bronchus, the guide sheath together with the R-EBUS probe was inserted through the working channel and advanced under fluoroscopic guidance. EBUS images before transbronchial sampling were categorized into 3 patterns: ‘within’ (the probe was located in a bronchus that was inside the lesion), ‘adjacent to’ (the probe was located in a bronchus that ran alongside the lesion), or ‘invisible’ (the probe was not able to reach the lesion) [8]. After locating the lesion, the probe was removed while the guide sheath was kept in place for subsequent sampling, also under fluoroscopic guidance. Transbronchial needle aspiration (TBNA) with or without a guide sheath was additionally performed when the operator deemed that the sample amount was insufficient [11, 13].

**Fig. 1:**
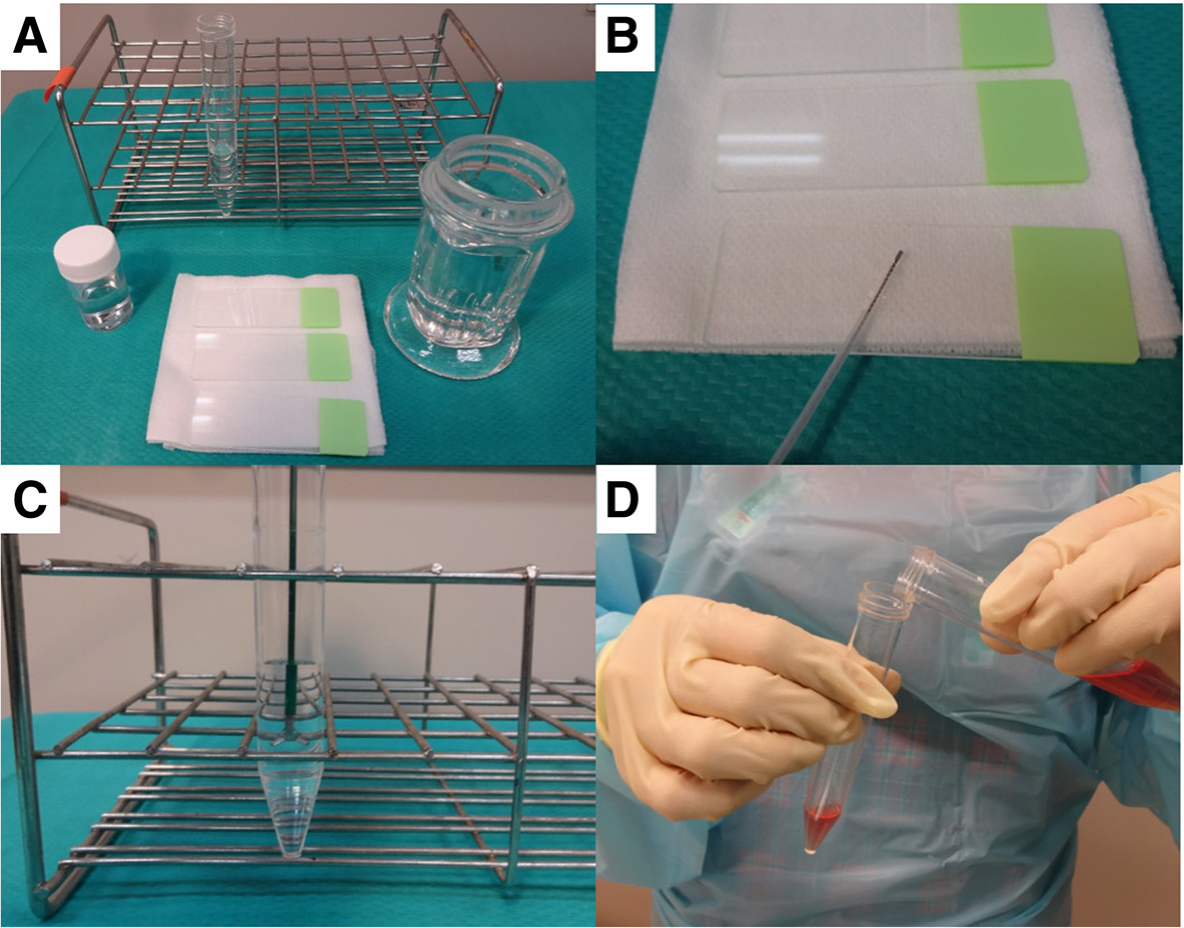
Specimen collection set for endobronchial ultrasound with a guide sheath (EBUS-GS). (**a**) A specimen collection set for EBUS-GS routinely consists of 10 % formalin (*lower left*), glass slides (*lower centre*), 95 % alcohol (*lower right*), and 5 mL normal saline (*upper*). (**b**) Brush and needle smears and tissue stumps on glass slides are sent for cytologic examination. (**c**) After each sampling, the brush, needles, and forceps are rinsed with 5 mL normal saline (device wash). (**d**) The device wash sample is divided into 2 test tubes, which are sent for cytologic and microbiologic examinations, respectively

Brush and needle smears and tissue stumps on glass slides were sent for cytologic examination. Tissue samples obtained by using forceps were sent for histopathologic examination. After each sampling, the brush, needles, and forceps were rinsed with 5 mL normal saline (device wash) [14]. After all the samplings, the guide sheath was removed and the lumen was flushed with the device wash liquid. Then, the device wash sample was divided into 2 test tubes, which were sent for cytologic and microbiologic examinations, respectively (Fig. 1). Routine microbiologic examination comprised Gram stain, acid-fast bacilli smear, polymerase chain reaction of *Mycobacterium tuberculosis* (TB), and cultures including TB. Although additional bronchial wash was performed in 2 patients, those results were not assessed in this study.

### Diagnostic criteria

Final diagnosis was determined based on either one or a combination of the results of EBUS-GS, EBUS-TBNA, surgery, or clinical follow-up for at least 3 months. A lesion was designated as inflammation if it showed no significant pathologic or clinical findings, and it decreased in size on follow-up CT.

For malignant lesions, class IV/V results on cytology or specific malignant findings on histopathology were considered as diagnostic. For benign lesions, specific benign findings on histopathology (e.g. necrotizing epithelioid granuloma, acute-angle branching hyphae, or inflammation) or positive cultures on microbiology were considered as diagnostic. Histopathologic results were reviewed by 2 investigators (MH and KK). Overall, EBUS-GS was considered as diagnostic when at least 1 of the cytologic, histopathologic, or microbiologic results was diagnostic.

### Complications of EBUS-GS

All bronchoscopies were performed in inpatient settings. After every procedure, chest radiography was performed and oral antibiotics (cefcapene pivoxil 300 mg/day for 3 days) were administered. A major complication was defined as an event that necessitated premature termination of the procedure or a symptomatic postprocedure sequela, including pneumothorax, haemorrhage, infection, air embolism, or other untoward life-threatening outcome [8].

### Statistical analysis

Descriptive statistics are presented as frequency, percentage, and median (range). Univariate analyses were performed by using Fisher’s exact test for categorical data. Multivariate logistic regression analysis was performed to determine the factors associated with increased diagnostic yield. Variables for the multivariate analysis model were chosen from the results of the univariate analyses and experimentally predicted factors, such as lesion size and bronchus sign. A 2-tailed *P* value of <0.05 was considered to indicate statistical significance. All statistical analyses were performed by using EZR (Saitama Medical Center, Jichi Medical University, Saitama, Japan), a graphical user interface for R (The R foundation for Statistical Computing, Vienna, Austria) [15].

## Results

**Fig. 2:**
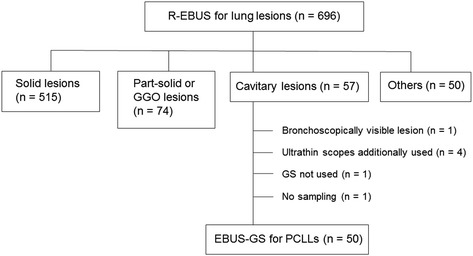
Study diagram. *Abbreviations*: *EBUS-GS* endobronchial ultrasound with a guide sheath, *GGO* ground-glass opacity, *GS* guide sheath, *PCLL* peripheral cavitary lung lesion, *R-EBUS* radial endobronchial ultrasound

Among 696 R-EBUS procedures performed between July 2013 and October 2015 at our institution, 50 PCLLs examined by using EBUS-GS were included in this study (Fig. 2). A summary of the baseline characteristics of the 50 patients is shown in Table 1. The study population had a median age of 67 years and mostly consisted of male patients. Median lesion size, cavity wall thickness, and distance from the costal pleura were 33.5, 11 , and 0 mm, respectively. Most lesions were located in the lower lobe (54 %), and bronchus sign was positive in the majority of cases (82 %). VBN was used for route planning in 27 patients (54 %).

**Table 1:** Baseline characteristics of patients with peripheral cavitary lung lesions

Variable	All (*n* = 50)
Age, median (range), year	67 (45–86)
Sex, *n* (%)	
Male	32 (64)
Female	18 (36)
Lesion size, median (range), mm	33.5 (16–100)
Cavity wall thickness, median (range), mm	11 (3–64)
Distance from costal pleura, median (range), mm	0 (0–22)
Lobar location, *n* (%)	
Upper	21 (42)
Middle	2 (4)
Lower	27 (54)
Bronchus sign, *n* (%)	
Positive	41 (82)
Negative	9 (18)
VBN, *n* (%)	
Yes	27 (54)
No	23 (46)
EBUS, *n* (%)	
Within	26 (52)
Adjacent to	18 (36)
Invisible	6 (12)

*Abbreviations*: *EBUS* endobronchial ultrasound, *VBN* virtual bronchoscopic navigation

Details of the final diagnoses are shown in Table 2. Among 27 malignant lesions, 21 were successfully diagnosed by using EBUS-GS (diagnostic yield, 77.8 %), 4 were diagnosed according to other examinations after EBUS-GS (EBUS-TBNA in 1 lung adenocarcinoma, surgery in 2 lung adenocarcinomas and 1 lung squamous carcinoma), and the remaining 2 were clinically diagnosed as lung cancer according to more than 6 months of CT follow-up and multidisciplinary team discussions. Among 23 benign lesions, 19 were successfully diagnosed by using EBUS-GS (diagnostic yield, 82.6 %), 3 were diagnosed according to other examinations after EBUS-GS (surgery in 1 nontuberculous mycobacteria [NTM], sputum culture in 2 NTMs), and the remaining 1 was clinically diagnosed as rheumatoid nodule based on its marked decrease in size by immunosuppressive therapy for rheumatoid arthritis. Overall, sensitivity, specificity, positive predictive value and negative predictive value for diagnosing malignancy were 77.8, 100, 100 and 79.3 %, respectively.

**Table 2:** Final diagnoses and diagnostic yields according to sample category

Final diagnosis	All (*n* = 50)	Diagnosed by EBUS-GS, *n* (%)	Diagnosed by cytologic sample, *n* (%)	Diagnosed by histologic sample, *n* (%)	Diagnosed by microbiologic sample, *n* (%)
Lung cancer	27	21 (77.8)	17 (63.0)	20 (74.1)	0 (0)
Adenocarcinoma	10	7 (70)	6 (60)	7 (70)	0 (0)
Squamous cell carcinoma	15	14 (93.3)	11 (73.3)	13 (86.7)	0 (0)
Clinically diagnosed	2	0 (0)	0 (0)	0 (0)	0 (0)
Benign	23	19 (82.6)	0 (0)	16 (69.6)	11 (47.8)
TB	3	3 (100)	0 (0)	3 (100)	3 (100)
NTM	12	9 (75)	0 (0)	6 (50)	8 (66.7)
Lung abscess	4	4 (100)	0 (0)	4 (100)	0 (0)
Aspergillosis	1	1 (100)	0 (0)	1 (100)	0 (0)
Rheumatoid nodule	1	0 (0)	0 (0)	0 (0)	0 (0)
Inflammation	2	2 (100)	0 (0)	2 (100)	0 (0)

*Abbreviations*: *EBUS-GS* endobronchial ultrasound with a guide sheath, *NTM* nontuberculous mycobacteria, *TB Mycobacterium tuberculosis*

The diagnostic yields for cytologic, histopathologic and microbiologic samples also are shown in Table 2. For 27 malignant lesions, 17 (63.0 %) cytologic and 20 (74.1 %) histopathologic samples were diagnostic. With respect to histologic type of lung cancer, squamous cell carcinoma was more diagnosable by histopathology than adenocarcinoma (86.7 vs 70 %). For 23 benign lesions, 16 (69.6 %) histopathologic and 11 (47.8 %) microbiologic samples were diagnostic. For 15 lesions with mycobacterial infection (TB in 3, NTM in 12), the sensitivity of EBUS-GS was 80 % (12/15), and 9 (60 %) and 11 (73.3 %) lesions were successfully diagnosed by using histopathologic and microbiologic samples, respectively. All of the other infectious diseases, including 4 lung abscesses and 1 Aspergillosis, were successfully diagnosed by histopathologic samples only.

The overall diagnostic yield for EBUS-GS was 80 % (40/50). Univariate analyses indicated that PCLLs in which the EBUS probe could be placed within the lesion had a significantly higher diagnostic yield compared with when the EBUS probe was adjacent to the lesion or invisible (92.3 vs. 66.7 %, *P* = 0.03). Multivariate logistic regression analyses using the possible cofounders of lesion size, bronchus sign and EBUS probe ‘within’ also revealed that the only significant factor affecting diagnostic yield was EBUS probe ‘within’ (odds ratio, 7.04; *P* = 0.03) (Table 3). Among the 50 procedures, pulmonary infection occurred in 1 patient (2.0 %). In this case, although fever and increased inflammatory reaction occurred after EBUS-GS, such symptoms were resolved by intravenous antibiotic therapy. No other major complications, including pneumothorax or significant haemorrhage that necessitated premature termination of the procedure, were reported.

**Table 3:** Factors affecting diagnostic yield of EBUS-GS for peripheral cavitary lung lesions

Variable	Univariate analysis		Multivariate analysis	
	Diagnostic yield, (%)	*P* value	OR (95 % CI)	*P* value
Age, year				
≥ 70	17/20 (85)	0.72	–	
< 70	23/30 (76.7)			
Sex				
Male	26/32 (81.3)	1	–	
Female	14/18 (77.8)			
Lesion size, mm				
> 30	25/31 (80.6)	1	1.19 (0.24–5.95)	0.83
≤ 30	15/19 (78.9)			
Cavity wall thickness, mm				
> 10	22/27 (81.5)	1	–	
≤ 10	18/23 (78.3)			
Distance from costal pleura, mm				
> 10	10/11 (90.9)	0.42	–	
≤ 10	30/39 (76.9)			
Lobar location				
Lower	21/27 (77.8)	0.74	–	
Upper/middle	19/23 (82.6)			
Bronchus sign				
Positive	32/41 (78.0)	0.67	0.26 (0.02–2.90)	0.27
Negative	8/9 (88.9)			
VBN				
Yes	22/27 (81.5)	1	–	
No	18/23 (78.3)			
EBUS image				
Within	24/26 (92.3)	0.03	7.04 (1.27–38.90)	0.03
Adjacent to/invisible	16/24 (66.7)			

*Abbreviations*: *CI* confidence interval, *EBUS* endobronchial ultrasound, *EBUS-GS* endobronchial ultrasound with a guide sheath, *OR* odds ratio, *VBN* virtual bronchoscopic navigation

**Fig. 3:**
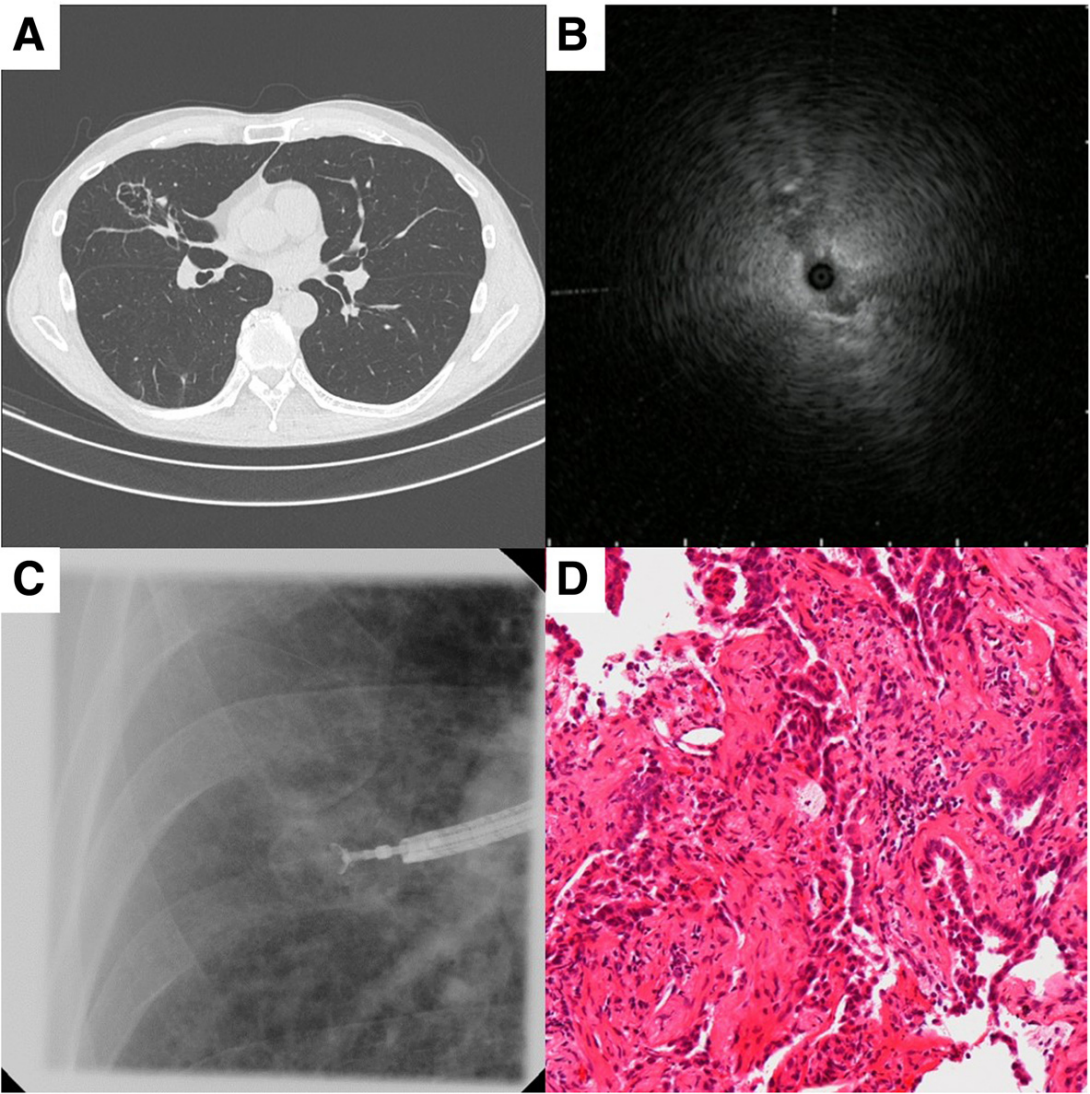
Case of a 64-year-old man with an abnormal shadow in the right middle lobe. (**a**) Computed tomography scan, showing a 32-mm peripheral cavitary lung lesion with a thin wall (3 mm). (**b**) Endobronchial ultrasound image is ‘adjacent to’ the lesion. (**c**) Fluoroscopic image during transbronchial biopsy through a guide sheath. (**d**) Histologic specimen obtained by using forceps, showing adenocarcinoma (hematoxylin and eosin stain, magnification ×200)

A representative case is shown in Fig. 3. The EBUS image before sampling was ‘adjacent to’ the lesion, which showed the thin wall of the lesion. With the guide sheath located just before the cavity wall, samples were obtained by using a brush and forceps. Cytologic and histologic specimens showed adenocarcinoma.

## Discussion

Although EBUS-GS has improved the diagnostic yield for peripheral lung lesions, reports on the use of EBUS-GS specifically for cavitary lesions are unavailable. To the best of our knowledge, this is the first report on the efficacy and safety of EBUS-GS for diagnosis of PCLLs. In addition, the utility of the practical sampling method of EBUS-GS for malignant and benign PCLLs was carefully evaluated in this study. Two important clinical observations were made. First, EBUS-GS had a high diagnostic yield (80 %) for PCLLs with a low risk of significant complications (2.0 %). Second, the EBUS-GS sampling method combined with cytologic, histopathologic, and microbiologic samples was effective for diagnosis of both benign and malignant PCLLs.

A cavity is the result of such pathologic processes as necrosis, cystic dilation of pulmonary structures, and displacement of lung tissue by cystic structures [16]. In addition, malignant lesions can become hollowed out due to treatment-related necrosis, internal cyst formation, or internal desquamation of tumour cells [17]. Active infectious and malignant diseases require a prompt and accurate diagnosis to minimize adverse outcomes and apply appropriate management. Although thin-section chest CT findings, such as cavity wall thickness, are helpful for diagnosis of cavitary lesions, sufficient pathologic or microbiologic samples are often necessary to ensure correct diagnosis [16]. TTNA is a useful nonsurgical method for examination of peripheral lung lesions, but its risk of complication is generally higher than TBB, and thus, it may have concerns about safety for cavitary lesions because of internal necrosis and inflammatory reactions [18, 19]. Actually, Zhuang et al. reported that although TTNA had an accuracy of 96.1 % for diagnosis of 102 cavitary lung lesions, complications included pneumothorax (8.8 %), alveolar haemorrhage (13.7 %), and hemoptysis (1 %) [20].

Currently, EBUS-GS is becoming a standard procedure for diagnosis of peripheral lung lesions based on its useful and safe profile. Therefore, confirmation of its diagnostic utility for PCLLs is also demanded in clinical practice. In the present study, we first reported a high diagnostic yield (80 %) for EBUS-GS for PCLLs. This result is comparable to the diagnostic yield for R-EBUS for peripheral lung lesions reported in recent studies. Therefore, EBUS-GS can be considered as a standard procedure for diagnosis of peripheral lung lesions and PCLLs [9, 21–23]. Moreover, we demonstrated that the diagnostic yield was better when the R-EBUS probe was within the lesion, which has been similarly shown in previous studies [24–26]. However, in the present study, no other important factors, such as bronchus sign, lesion size, or cavity wall thickness, were found to be associated with increased diagnostic yield, despite the small sample size. Especially in larger lesions, the inflammatory reaction around the cavity wall might interfere with obtaining diagnostic samples even if bronchus sign is positive. A previous study on the use of TTNA for diagnosis of cavitary lung lesions reported that more nondiagnostic samples were obtained from lesions with thin walls [20]. Repeated biopsies at precise locations might contribute to accurate diagnosis even for PCLLs with thin walls, as shown in the representative case.

The complication rate in the present study was 2.0 % (pulmonary infection), which is lower than that reported in previous studies on the use of TTNA for diagnosis of cavitary lung lesions [3, 20]. There was no case of pneumothorax or significant haemorrhage, which are major complications of TBB for peripheral lung lesions. Precise localisation by R-EBUS and the tamponade effect of the guide sheath might reduce the risk of complications, even for PCLLs. Although TBB using R-EBUS has been reported as a safe procedure for diagnosis of peripheral lung lesions, a previous study showed that among 965 peripheral lung lesions examined by using EBUS-GS, pulmonary infection occurred in 5 patients (0.5 %), 2 of which had PCLLs [8]. Therefore, although EBUS-GS was found to be safe for diagnosis of PCLLs in the present study, we should be mindful of the risk of pulmonary infection.

This study also demonstrated that the EBUS-GS sampling method combined with cytologic, histopathologic, and microbiologic samples was effective for diagnosis of both benign and malignant PCLLs. In the present study, diagnostic tissue samples were obtained by using TBB for 74.1 % of malignant lesions and 69.6 % of benign lesions. Recently, histopathologic diagnosis of lung cancer is becoming important for definitive treatment including chemotherapy and tyrosine kinase inhibitors [27]. Moreover, because cavitary lung cancer has a worse prognosis than non-cavitary lung cancer, successful histopathologic diagnosis using a safe technique is demanded [28]. This should also be applied to benign cavitary lesions. Chan et al. recently reported that necrotizing granulomatous inflammation was demonstrated by using EBUS-GS in 10 of 22 patients with pulmonary TB, and that histopathologic diagnosis of TB might contribute to prompt treatment [9]. Certainly, histopathologic diagnosis is necessary for appropriate treatment not only for malignant lesions but also for benign lesions. Even for PCLLs, EBUS-GS has the advantages of locating lesions accurately, controlling bleeding, and obtaining adequate tissue samples.

In the present study, microbiologic samples demonstrated a diagnostic yield of 47.8 % for benign diseases; the diagnostic yield was particularly high for mycobacterium infection, which is the main cause of a cavitary lung lesion (100 % in TB, 66.7 % in NTM). Microbiology results were negative for infectious cases with lung abscess or Aspergillosis, though anaerobic or *Aspergillus* culture was not performed routinely. Because the culture sensitivities of these diseases using bronchoscopy samples are not satisfactory [29, 30], routine bronchial wash or bronchoalveolar lavage after removing the guide sheath may not be necessary. Instead, obtaining adequate tissue samples can contribute to definitive treatment. Although the guide sheath has to be introduced into the lesion for adequate tissue sampling, the R-EBUS probe was located within the lesion in only 26 patients (52 %) in the present study, despite a positive bronchus sign in the majority of cases (82 %). Although this may be due to a lack of skills in the trainees, additional TBNA may be important for improved diagnosis of PCLLs, especially when they are not located by using R-EBUS [13, 31–33].

The present study has several limitations. First, this was a retrospective, nonrandomised, single-institution study with a small sample size. Second, the procedures were not performed by the same operator. Thus, it remains unclear whether the results can be generalized to other institutions.

## Conclusions

In conclusion, we presented the first report on EBUS-GS for PCLLs based on our clinical experience. Our results indicate that EBUS-GS is a safe and effective procedure for diagnosis of PCLLs. Sufficient cytologic, histopathologic, and microbiologic samples can be obtained for both benign and malignant PCLLs. Further multicenter prospective studies are recommended in the future.

### Ethics approval and consent to participate

The Institutional Review Board of Osaka Prefectural Medical Center for Respiratory and Allergic Diseases approved this study without the need to obtain informed consent.

### Availability of data and materials

The dataset supporting the conclusions of this article is presented within the article. The detailed clinical data set is not publically available to protect research subject privacy and confidentiality.

## Abbreviations

CT: computed tomography
EBUS: endobronchial ultrasound
EBUS-GS: endobronchial ultrasound with a guide sheath
GGO: ground-glass opacity
NTM: nontuberculous mycobacteria
PCLL: peripheral cavitary lung lesion
R-EBUS: radial endobronchial ultrasound
TB: *Mycobacterium tuberculosis*
TBB: transbronchial biopsy
TBNA: transbronchial needle aspiration
TTNA: transthoracic needle aspiration
VBN: virtual bronchoscopic navigation
